# Cdx ParaHox genes acquired distinct developmental roles after gene duplication in vertebrate evolution

**DOI:** 10.1186/s12915-015-0165-x

**Published:** 2015-08-01

**Authors:** Ferdinand Marlétaz, Ignacio Maeso, Laura Faas, Harry V. Isaacs, Peter W. H. Holland

**Affiliations:** Department of Zoology, University of Oxford, South Parks Road, Oxford, OX1 3PS UK; Department of Biology, University of York, Heslington, York, YO10 5DD UK; Present address: Centro Andaluz de Biología del Desarrollo (CABD), Consejo Superior de Investigaciones Científicas/Universidad Pablo de Olavide, Sevilla, Spain

**Keywords:** Cdx, Gene expression, Paralogues, Posterior axial patterning, Transcriptomics, Vertebrates, Whole genome duplication

## Abstract

**Background:**

The functional consequences of whole genome duplications in vertebrate evolution are not fully understood. It remains unclear, for instance, why paralogues were retained in some gene families but extensively lost in others. Cdx homeobox genes encode conserved transcription factors controlling posterior development across diverse bilaterians. These genes are part of the ParaHox gene cluster. Multiple Cdx copies were retained after genome duplication, raising questions about how functional divergence, overlap, and redundancy respectively contributed to their retention and evolutionary fate.

**Results:**

We examined the degree of regulatory and functional overlap between the three vertebrate Cdx genes using single and triple morpholino knock-down in *Xenopus tropicalis* followed by RNA-seq. We found that one paralogue, *Cdx4*, has a much stronger effect on gene expression than the others, including a strong regulatory effect on FGF and Wnt genes. Functional annotation revealed distinct and overlapping roles and subtly different temporal windows of action for each gene. The data also reveal a colinear-like effect of Cdx genes on Hox genes, with repression of Hox paralogy groups 1 and 2, and activation increasing from Hox group 5 to 11. We also highlight cases in which duplicated genes regulate distinct paralogous targets revealing pathway elaboration after whole genome duplication.

**Conclusions:**

Despite shared core pathways, Cdx paralogues have acquired distinct regulatory roles during development. This implies that the degree of functional overlap between paralogues is relatively low and that gene expression pattern alone should be used with caution when investigating the functional evolution of duplicated genes. We therefore suggest that developmental programmes were extensively rewired after whole genome duplication in the early evolution of vertebrates.

**Electronic supplementary material:**

The online version of this article (doi:10.1186/s12915-015-0165-x) contains supplementary material, which is available to authorized users.

## Background

It has long been postulated that duplication of genes generates new genetic material on which natural selection can act, and hence that gene duplication might facilitate the evolution of new characters (for example [1–3]; reviewed by [4]). A refinement of this model postulates that gene duplication permits complementary mutations in daughter genes or their regulatory elements, and this in turn releases genes from evolutionary constraint according to the duplication-degeneration-complementation model [5]. Duplications can involve single genes or sets of linked genes, or they can derive from polyploidy events in which the entire genome is duplicated. A classic example that has attracted much attention occurred early in the evolution of vertebrates. There is good evidence from gene family and synteny analyses that two whole genome duplications (WGDs) occurred in the vertebrate lineage, after its divergence from tunicates and cephalochordates, but before the diversification of jawed vertebrates and possibly even before divergence of lampreys [6–10]. The widespread interest in these two round (2R) genome duplications revolves around the possibility that the generation of thousands of new genes may have facilitated the evolution of the complex vertebrate body plan. Our own existence may therefore have been dependent on genome duplication. This hypothesis is hard to test, however, since one is asking whether it would have been possible for vertebrates to have arisen without genome duplications. Unable to rewind evolution, indirect tests must be proposed.

There can be extensive gene loss following genome duplication, such that only a proportion of genes are retained in multiple copies. Using the amphioxus genome as a reference, Putnam et al. [10] detected a significant skew in the functional categories of genes retained as multiple copies in vertebrates following the two WGD events, compared to those that reverted to single copy. Genes involved in developmental processes, cell signalling, cell communication, and neurobiology, as well as genes encoding transcription factors were preferentially retained as multiple copies. Although there may be taxonomic differences in patterns of gene loss, the result is consistent with pre-genomic analyses, since the first strong evidence for gene duplication in vertebrate ancestry came from analyses of homeobox and other developmental genes, and not from housekeeping genes (reviewed by [11, 12]). Transcription factors, such as those encoded by homeobox genes, are often at important nodes in gene regulatory networks controlling subsidiary modules dependent on cellular context. Several models have been proposed for the evolution of gene regulatory networks and modules after WGD; for example, entire modules could remain redundant because of dosage balance constraints or to provide developmental robustness, or alternatively networks could be rewired to effect novel biological functions [13]. One possibility, therefore, is that developmental programs were elaborated following genome duplication, with duplicate genes encoding transcription factors being recruited into the distinct roles required to pattern the complex vertebrate embryo.

**Fig. 1:**
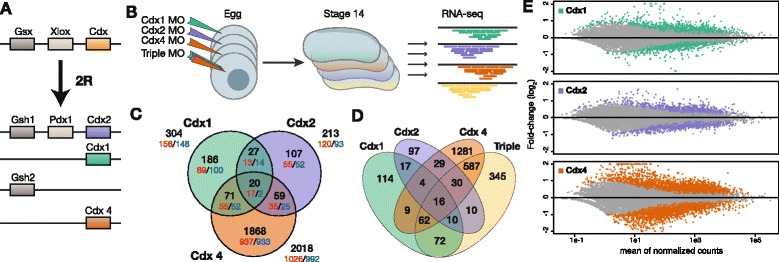
Transcriptomic assessment of Cdx paralogue function during *Xenopus tropicalis* development. **a** Duplication of the ancestral ParaHox cluster and subsequent gene loss in vertebrates resulting in three Cdx genes. **b** Experimental procedure: injection of eggs with morpholino oligonucleotides (MO) targeting specific Cdx copy, collection of stage 14 (early neurula) embryos for RNA extraction, and transcriptomic characterisation by RNA-seq. **c** Gene set overlap of the three Cdx MO targets inferred by differential expression analysis of RNA-seq data (see Methods). Venn diagram categories are specified for all genes (black), upregulated genes (red), and down-regulated genes (blue). **d** Gene set overlap for three Cdx MOs and co-injection of all three MOs (triple). **e** Gene expression and fold-change intensities across differential expression for all three Cdx MOs. Above scale fold-change values are noted as triangles. This representation reveals a strong quantitative extent of *Cdx4* MO effect compared with *Cdx1* and *Cdx2* MOs

To investigate this possibility, we sought a family of homeobox genes retained in multiple copies after vertebrate 2R WGD, and with similar gene family composition in different vertebrate species. The Cdx gene family, homologous to the *Drosophila* gene *caudal* (*cad*), fulfils these criteria. In the ancestor of vertebrates, there was a single Cdx gene, chromosomally close to two other homeobox genes, Pdx and Gsx; the cephalochordate *Branchiostoma floridae* retains this condition [14]. After two rounds of whole genome duplication and some gene loss, the typical condition seen for jawed vertebrates is three Cdx genes, one Pdx, and two Gsx (Fig. 1a). This composition is seen in human, mouse, chicken, *Xenopus tropicalis*, and many other diploid species [15, 16]. In each case, the same three Cdx genes are present, named *Cdx1*, *Cdx2*, and *Cdx4*; there is no *Cdx3* gene. Teleost fish also have three Cdx genes despite having undergone an additional WGD; these are two copies of *Cdx1* (generated in the teleost fish 3R event) and a single *Cdx4* [15]. The consistency of the threefold condition for the Cdx genes, maintained over hundreds of millions of years of evolution, attracts us to Cdx genes as a useful case for analysing gene retention. The vertebrate Cdx genes are also interesting since they have been implicated in a range of developmental processes, notably specification of posterior identity along the body axis, activation of Hox genes, differentiation of epithelial cell fates in the gut, and haematopoiesis [17–21].

In *X. tropicalis*, as in other vertebrates, the three Cdx genes have slightly different spatiotemporal expression patterns. Although each gene is expressed in the posterior part of the body, *Cdx4* has a more anterior expression limit in the neural tube, and *Cdx1* and *Cdx2* expression persists longer than *Cdx4* in the posterior gut endoderm [22]. The absence of radical differences between expression patterns is intriguing, apparently challenging the idea that elaboration of developmental roles explains the retention of the three Cdx genes over 500 million years of vertebrate evolution. To investigate the degree of functional overlap, we have previously used injection of translation-blocking MOs to interfere with action of each Cdx gene individually and in combination during *X. tropicalis* development [23]. These experiments revealed that the three genes have largely additive roles in the posterior of the developing embryo. For example, MO knockdown of each Cdx gene individually gave a similar range of posterior defects, including a highly penetrant axis truncation phenotype. Knockdown of all three genes together was more effective than blocking translation of any individual Cdx gene, and intriguingly a truncation defect caused by disruption of *Cdx1*, *Cdx2*, and *Cdx4* together is largely rescued by injection of RNA coding for a synthetic transcriptional activator containing the *Cdx4* DNA binding domain [23]. Together, these experiments suggest that the three genes are involved in many of the same biological pathways in the posterior of the developing embryo, and that it is an overall landscape of Cdx activity that is necessary. The question remains on why three Cdx genes are necessary to generate this landscape of activity and, more generally, why many vertebrate WGD gene paralogues have been retained for 500 million years even in the apparent absence of strong expression patterns and functional differences. One hypothesis is that functional overlap gives robustness to developing systems, especially in the face of environmental or stochastic variation, and thus redundancy can be selected for [24].

The possibility we wished to address in the current study is whether there are functional differences between WGD paralogous genes that may be masked by their shared roles and expression domains such as the shared role for Cdx genes in patterning the posterior body. To investigate this, and gain further insight into why these three duplicate genes have been retained in evolution, we carried out gene disruption followed by RNA-seq gene expression profiling. We deployed the same MO disruption strategy as used previously, since this approach has been well characterised for these genes [23]. We then examined downstream effects on *X. tropicalis* gene expression using RNA-seq. Our analyses reveal shared genes and biological pathways under the control of all Cdx genes, but each Cdx gene is implicated in a suite of specific biological pathways. This implies that sub- and neo-functionalization have altered transcription factor targets as well as regulatory network architecture after vertebrate WGDs.

## Results

### Striking quantitative differences in downstream targets of *Cdx1*, *Cdx2*, and *Cdx4*

**Fig. 2:**
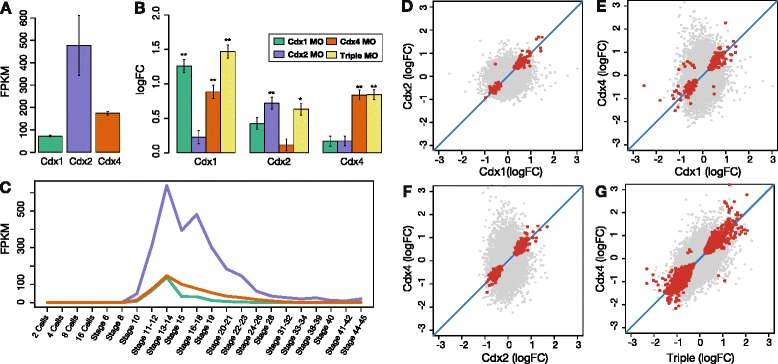
Cdx expression, cross-regulation, and pair-wise comparison. **a** Normalized expression of three Cdx paralogues at stage 14 derived from the control uninjected embryos. **b** Fold-change effect of alternative Cdx MOs on Cdx expression. Error bars indicate standard error; significance level noted as (**) and (*) for Benjamini-Hochberg-adjusted *P* <0.005 and <0.05, respectively. **c** Temporal expression profile of Cdx paralogues recovered from data of [25]. **d–g** Pairwise comparison of gene-specific fold-change triggered by distinct Cdx MOs. Genes with expression affected by both Cdx MOs are plotted as red dots, other genes plotted as grey dots

We used injection of translation-blocking morpholino oligonucleotides (MOs) in *X. tropicalis* to investigate whether, or to what extent, duplicate genes of the Cdx homeobox gene family have distinct effects on developmental pathways in the embryo, assessed through alterations to transcriptional profiles (Fig. 1b). In *X. tropicalis*, all three Cdx genes are initially expressed in the early mesoderm at the start of gastrulation (stage 10) [22]. Following the end of gastrulation, the Cdx genes are expressed in both the ectoderm and mesoderm in the posterior of the neurula stage embryo (stage 14). We chose the early neurula stage, 6 hours after the initiation of Cdx expression, for transcriptomic analyses because it represents the peak of Cdx expression (Fig. 2c) and the stage when Hox genes, known targets of Cdx, are activated. Replicate sets of embryos were injected with a standard control morpholino or MOs targeted against the *Cdx1*, *Cdx2*, and *Cdx4* mRNAs, either individually or in combination (Fig. 1b). After culturing to stage 14, injected and uninjected control embryos were collected [23] and Illumina RNA-seq used to profile gene expression. As previously reported [23], no large scale disruption of anteroposterior organisation is apparent by this stage.

Differential expression analyses were applied to evaluate the effect of each duplicate Cdx gene on the embryonic transcriptome. Our approach is expected to identify direct targets of the Cdx transcription factors, and also indirect targets and modulated downstream pathways. Using mock-MO injection as a reference, we observed a strong impact of Cdx morpholino injection on subsequent gene expression at stage 14, with numbers of differentially expressed genes ranging from 461 to 3471 across conditions (adj. *P* <0.05; Additional file 1: Table S1). To obtain a more restricted list of unambiguously regulated genes, we applied more conservative filters based on expression and fold-change levels and accounting for injection effect (see Methods and Additional file 1: Table S1).

Using these criteria, we found a striking difference in the respective effect of each Cdx gene knockdown on gene expression: *Cdx4* has a much stronger biological effect (2018 differentially expressed genes), than its paralogues *Cdx1* and *Cdx2* (304 and 213 regulated genes, respectively). In *Cdx1* and *Cdx2*, approximately half of the differentially expressed genes belong to a core set of 177 genes that are affected by at least two Cdx MOs, but only 20 genes are co-regulated by all three Cdx MOs (Fig. 1c). The proportion of up- and down-regulated genes is comparable in each condition, including for the genes affected in more than one condition (Fig. 1c). The stronger *Cdx4* effect also extends to the intensity of the fold-change observed for significant genes: 362 genes show a change in expression level greater than twofold in *Cdx4* MO embryos, whereas only 42 and 11 genes reach twofold change in *Cdx1* and *Cdx2* MO embryos (Fig. 1e). This effect is also consistent with the higher *P* values in differential expression for *Cdx4* MO treatment (Additional file 2: Figure S2).

Through triple injection of all three Cdx MOs, we examined the effect of interfering with the complete Cdx gene family (Fig. 1d). We found 1132 genes differentially expressed after triple MO injection using the same cut-offs. While most of these targets (787 genes) were also detected in the sets of genes affected by single Cdx MOs, 345 genes were interestingly found to be differentially regulated only when all three Cdx are disrupted (Fig. 1d). The detection of this target set suggests that a combination of all three Cdx genes is necessary for the activation of some pathways, or alternatively that paralogous Cdx genes can compensate for each other in the regulation of these genes. We found that the 345-gene co-regulated set includes genes involved in several developmental processes (Additional file 3: Table S2). We also note that some genes affected by single MOs (particularly *Cdx4* MO) are not affected by the triple MO. The likely explanation is that some targets require a higher dose of MO to effect statistically significant change in expression (to maintain the same total MO dose, one third amounts were used in the triple MO).

### Distinct downstream effects of paralogous Cdx genes

A major goal of this study was to determine the degree of functional difference between the three duplicate Cdx genes in the vertebrate embryo. We therefore tested whether the observed effects are attributable to the biological properties of each Cdx gene, or to experimental or quantitative effects.

We first determined that all Cdx genes show comparable temporal expression profiles in normal conditions using our data and that of Tan et al. [25]. All three genes peak in expression at stage 14; however, we note that *Cdx2*, not *Cdx4*, is the most abundant transcript (Fig. 2a, c), even though *Cdx4* disruption affects a larger number of target genes. We then considered the respective MO effects on Cdx gene expression to verify specificity (Fig. 2b). Each Cdx morpholino increases the abundance of its targeted transcript to a comparable degree for all three Cdx MOs (Fig. 2b). A similar effect has been noted before in other studies using translation-blocking MOs, and may reflect transcript stabilisation or compensation mechanisms [26]. We also note a significant up-regulation of *Cdx1* after *Cdx4* MO injection, suggesting that *Cdx4* represses *Cdx1.*

We searched for evidence of distinct downstream effects by evaluating the effect of distinct pairs of Cdx MOs on gene expression (Fig. 2d–g). For each of these comparisons, we plotted expression fold-change for genes significantly affected by both MO treatments (red dots), and genes that were unaffected by treatment or affected by only one MO. It is notable that the global transcriptome response can be skewed by specific MO effects. Thus, comparison of *Cdx1* and *Cdx2* MO (Fig. 2d) gives a symmetric cloud of points, whereas the strong effect of *Cdx4* MO noticeably shapes the cloud of points by activating or repressing genes that are unaffected by *Cdx1* or *Cdx2* MO (Fig. 2e, f). Conversely, co-regulated genes display similar fold-change response in both conditions, as shown by close fitting to the diagonal line in each graph (Fig. 2d–g). These observations indicate that the distinctiveness of *Cdx4* is driven by presence of additional targets, not differential response of co-regulated targets. Disruption of *Cdx4* gives a transcriptional response of comparable intensity to disruption of all three genes, compatible with the larger quantitative effect of *Cdx4* (Fig. 2g).

**Fig. 3:**
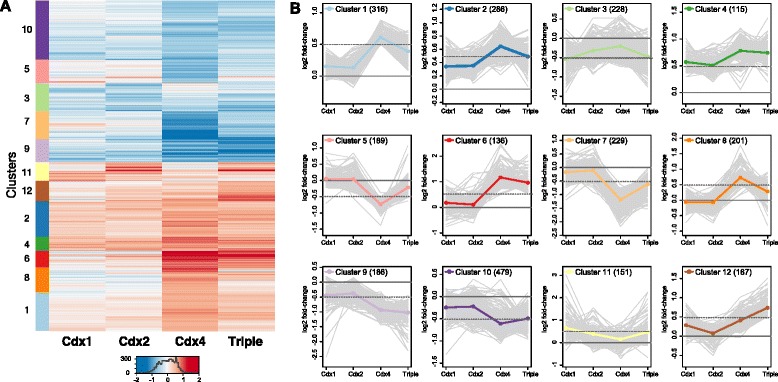
K-mean clustering of multiple Cdx MOs effects. **a** Heatmap representation of gene expression fold-changes triggered by the three Cdx MOs and the co-injection triple MOs. Genes are arranged according to k-mean clustering and 12 clusters (left) are delineated to capture the diversity of responses to treatments. **b** Detail of gene expression response to Cdx MOs in the 12 selected clusters. The average response is plotted as a bold line while response of each member gene of the cluster is plotted in a thin grey line

To identify differentially regulated gene sets, we applied a clustering procedure on expression fold-change in the distinct MO treatments, using all genes that are differentially expressed in at least one condition (Fig. 3). These analyses revealed diverse gene sets with markedly different response pattern to distinct Cdx MOs. For example, genes in clusters 6 and 8 are strongly up-regulated by disruption of *Cdx4* function, but not at all are affected by *Cdx1* or *Cdx2* disruption. Similarly, cluster 5 and 7 genes show specific down-regulation with *Cdx4* disruption. In contrast, clusters 1, 2, 9, and 10 are up- or down-regulated by disruption of any of the three Cdx genes, albeit more strongly by *Cdx4* disruption. Cluster 12 genes respond to interference of *Cdx1* and *Cdx4*, but not *Cdx2*; cluster 11 genes are affected by *Cdx1* and *Cdx2* but not *Cdx4*. We examined functional categories associated with these diverse responses by performing term enrichment analyses on each of these gene sets (Additional file 4: Table S3). We found clear functional distinctiveness of some clusters: cluster 1 is closely associated with Wnt signalling (14 genes, *P* = 0.03), cluster 2 is enriched in genes involved in embryo development (12 genes, *P* = 0.01), and cluster 8 in chromatin organization-related genes (230 genes, *P* = 0.009). While most clusters are enriched in one or a few terms, cluster 9 captures several specific developmental processes, such as angiogenesis and digestive tract development, that are classically associated with Cdx function.

In summary, these analyses reveal that the relatively large effect of *Cdx4* is not solely a quantitative effect on shared targets, and that qualitatively different effects are caused by disruption of each of the three Cdx genes in development. We also uncover overlapping roles for the three Cdx genes in Wnt signalling, angiogenesis, and gut development.

### Categories of gene function affected by Cdx gene disruption

**Fig. 4:**
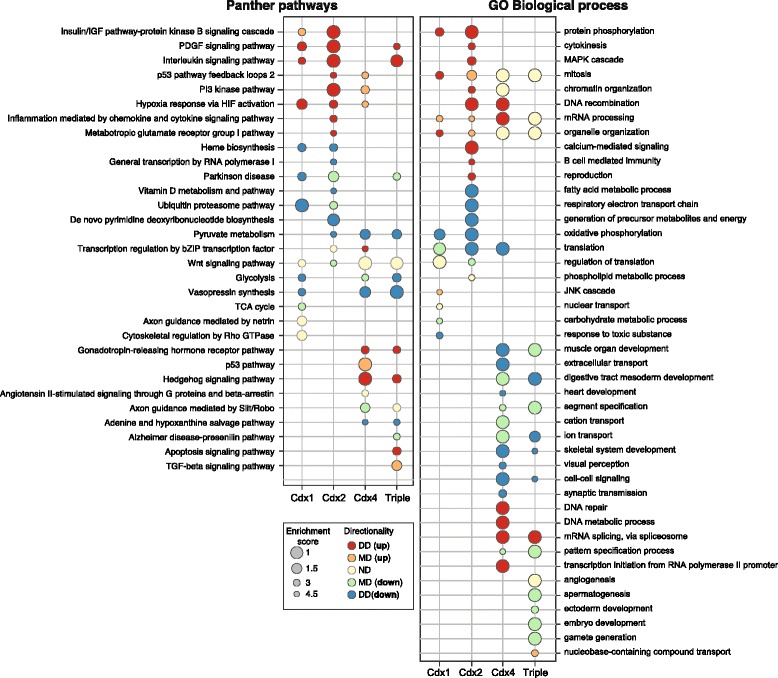
Gene set enrichment analysis of Cdx MO effects. Functional annotation derived from Panther pathways and the GO biological process version of Panther database were employed for term enrichment analysis using distinct tests accounting for direction of expression change: distinct directional (DD), mixed directional (MD), non-directional (ND), as well as UP or DOWN regulation. The scheme yielding the best enrichment score was retained as the one providing the best description of the enrichment for the term (bubble fill colour). Displayed terms were retained as showing an enrichment >5 in at least one condition

To further evaluate the degree of functional divergence between the Cdx paralogues, we conducted an enrichment analysis using gene sets derived from biological annotation: Panther pathways and Gene Ontology Biological Processes (Fig. 4). These analyses measure the consistency of significance statistics (*P* value) and gene expression variation (fold-change) over a gene set corresponding to a given annotation term. We identified important pathways and biological functions, such as Wnt signalling pathways (Panther) and ‘mitosis’ processes (GO Biological Processes), that are represented in gene sets regulated by all three paralogues. Such broad terms often do not exhibit a comprehensive up- or down-regulation, most likely because they encompass genes with antagonist interactions within the same pathway (e.g. intracellular effectors). Few annotation terms show such a shared enrichment, while many other pathways or biological processes appear regulated by one or two paralogues only. For instance, the genes affected by *Cdx1* and *Cdx2* disruption are enriched in down-regulated members of the ‘heme biosynthesis’ pathway (Fig. 4), consistent with a role of Cdx genes in blood cell specification [27]. Similarly, these same two Cdx genes are involved in the repression of the PDGF signalling pathway, as their disruption causes up-regulated expression of genes belonging to this category (Fig. 4). Enrichment analysis indicates only limited functional overlap of *Cdx4* with *Cdx1* and *Cdx2*, with several pathways seemingly only affected by *Cdx4*; these include Hedgehog signalling, the Gonadotropin pathway, and Slit/Robo axon guidance (Fig. 4). Finally, the triple morpholino treatment recapitulates the enrichment effects observed for individual Cdx genes in most cases. However, a few pathways, such as TGF-beta signalling, are only detected when all three Cdx are disrupted simultaneously (Fig. 4). These categories could correspond to gene sets of which individual members are regulated by distinct Cdx genes, and which only pass significance threshold when all members are activated simultaneously. Alternatively, these gene sets could reflect functional redundancy between co-expressed Cdx genes, or compensatory regulation after disruption of any one Cdx gene.

### Cdx genes effects on Hox genes differ according to genomic position

The role of Cdx genes in axial elongation and positional specification is mediated, at least in part, through activation of posterior Hox genes [19, 28, 29]. However, the effect on entire Hox gene clusters has not been examined.

**Fig. 5:**
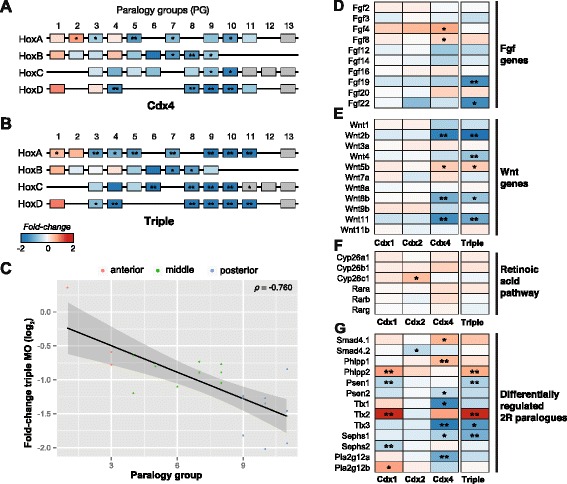
Distinct Cdx effect on known targets and 2R paralogues. Fold-change in expression induced by different Cdx MOs, or by triple Cdx MO injection, is indicated using a blue (down) to red (up) colour scale for each gene. **a**, **b** Schematized Hox gene clusters in *X. tropicalis* showing effects of Cdx gene knockdown on expression levels. Effects of *Cdx4* (**a**) and triple injection (**b**) shown. Genes in grey are below expression cut-off (FPKM<2). **c** Fold-change in Hox gene expression caused by triple MO injection plotted against paralogy group assignment; each data point represents one Hox gene. Only genes with statistically significant change in expression are included. Colours denote anterior (red), middle (green), and posterior (blue) paralogy group assignments, assigning group 3 to anterior. **d**, **e** Heatmap representation of MO effect on Cdx target gene pathways: Fgf genes, Wnt genes, Retinoic acid pathway. Only genes expressed at stage 14 (FPKM>2) are included. **g** Effect of Cdx genes on pairs or triplets of genes originated through 2R vertebrate genome duplication (see Methods) which show differential regulation by distinct Cdx paralogues. Significance of differential expression is denoted by one (*P* <0.05) or two (*P* <0.005) asterisks (**a**, **b**, **d**–**g**)

We investigated the effect of each Cdx MO on expression of Hox genes. We found that *Cdx4* regulates genes from all four Hox clusters, including a major effect on posterior Hox genes which are strikingly down-regulated by disruption of *Cdx4* function (Fig. 5a). Disruption of all three Cdx genes has an even stronger effect on Hox gene expression, with clear down-regulation of genes from paralogy group 5 through to paralogy group 10 or 11. At the other end of the Hoxa cluster, *Hoxa1* and *Hoxa2*, the most anteriorly-expressed Hox genes in the embryo, respond in the opposite direction, being up-regulated by disruption of Cdx gene function (Fig. 5a, b). Other anterior genes are affected more weakly by the triple MO or by *Cdx4*, while disruption of *Cdx1* or *Cdx2* may have similar effects, although these are weak and not verified statistically (Additional file 5: Figure S3).

To determine if these expression shifts are consistent with a trend across the Hox clusters, we assessed the relationship between expression fold-change and Hox gene paralogy group for the triple Cdx MO experiment, revealing a negative slope and a significant correlation (Spearman *ρ* = −0.76 and *P* = 6.3 × 10^−5^ when only significantly changing genes included, Fig. 5c; *ρ* = −0.79 and *P* = 8.9 × 10^−8^ when all points included, Additional file 5: Figure S3). To test more robustly the relationship between Hox gene identity and MO effect, we grouped Hox genes into three evolutionary and functional categories: anterior (PG1-3), middle (PG4-8), and posterior (PG9-13) paralogy groups. We determined that the expression fold-change caused by Cdx triple MO on anterior genes is significantly greater than that on middle genes (*P* = 0.0068), and greater for middle than posterior genes (*P* = 0.0007; one-tailed Kolmogorov–Smirnov test; Additional file 5: Figure S3).

### Signalling pathways

The *X. tropicalis* genome has 24 Wnt genes [30], of which 11 are expressed in our stage 14 RNAseq data (FPKM>2). Wnt genes are implicated in patterning of the vertebrate body axis, through both canonical and non-canonical pathways [31, 32], and are thought to be important effector genes from Cdx gene activity [23]. We found that disruption of *Cdx4* function, or of all three Cdx genes together, caused up-regulation of *Wnt5b* and down-regulation of *Wnt2b*, *Wnt8b*, and *Wnt11* (Fig. 5e and Additional file 6: Figure S4). *Wnt3a*, a reported downstream target of Cdx activity [33], was not affected in this study; *Wnt5A*, another previously reported target, is not covered by the current genome assembly and was not assessed [30]. Disruption of *Cdx1* and *Cdx2* activity did not result in clear effects on individual Wnt gene expression, although we note the generic effect on the Wnt gene pathway mentioned above. Together, these results are consistent with the *Cdx4* gene in normal development having a generally activating effect on Wnt activity, although the different responses of each Wnt gene suggest more subtle effects and that each Wnt gene should not be treated as equivalent.

Fibroblast growth factor (FGF) and retinoic acid signalling are also implicated in Cdx gene function [34], with a key role for FGF in activating Cdx genes in mesoderm [35–37]. Effects of Cdx genes on FGF genes have not previously been shown directly, although it is known that Cdx genes and FGF signalling cooperate in patterning the posterior spinal cord [38]. Our analyses indicate that expression of *FGF4* and *FGF8* genes is repressed by *Cdx4* (up-regulated by MO *Cdx4*; Fig. 5d), while, conversely, *FGF19* expression is activated (significant down-regulation by triple MO; Fig. 5d and Additional file 6: Figure S4). We did not detect direct effects on expression of genes in the retinoic acid signalling pathway, apart from a repressive effect of *Cdx2* on one of the cytochrome p450 genes involved in retinoic acid degradation, *Cyp26c1* (up-regulated by MO *Cdx2*; Fig. 5f).

### Temporal windows of Cdx function

**Fig. 6:**
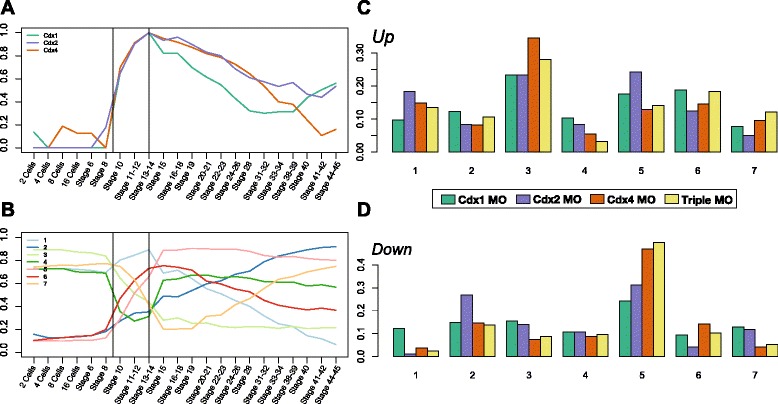
Temporal profile of Cdx expression and regulation. **a** Normalized expression of the three Cdx paralogues across the developmental time-course. **b** Transcriptome dynamic across development summarized in seven clusters recapitulating distinct temporal expression profiles using Euclidean distance K-mean clustering. Distribution of Cdx target genes across the seven temporal clusters for the up- (**c**) and down- (**d**) regulated genes

Recent reports have argued that gene expression is temporally structured in multiple waves in which key transcription factors play a major activating role [39]. We attempted to determine whether some of the differences in the gene sets regulated by Cdx paralogues were attributable to distinct temporal windows of activity during the developmental time course. All three Cdx genes reach their expression peak at stage 14, the stage investigated in this study (Figs. 2c and 6a); this is followed by a progressive decrease in expression, comparable in *Cdx2* and *Cdx4* but steeper for *Cdx1*. We categorized Cdx-regulated genes into seven clusters according to their temporal expression profiles, which distinguishes maternally expressed genes (notably cluster 3) from those initially activated at the mid-blastula transition (MBT) at stage 8 (clusters 2, 5 and 6; Fig. 6b). The proportion of genes regulated by each Cdx gene in each of the temporal clusters is broadly comparable (Fig. 6c, d). We note that all three Cdx genes play a role in repressing the expression of primarily maternal genes after the MBT (cluster 3, up-regulated by MO treatment; Fig. 6c). All three genes, but especially *Cdx4*, also play roles in activation of zygotic genes whose expression starts at the MBT (cluster 5, down-regulated by MO treatment; Fig. 6d). In addition, *Cdx2* plays a distinct role in activating genes with later expression onset (cluster 2, MO down-regulation; Fig. 6d), and repressing some post-MBT zygotic genes (cluster 5, MO up-regulation; Fig. 6c). In summary, examination of temporal expression profiles of Cdx gene targets did not suggest that the stronger *Cdx4* effect is related to a distinct activation timing of Cdx genes. Instead, this profiling reveals a shared role in modulating gene expression immediately after the MBT, coupled with subtle differences in the maintenance of a dynamic transcriptome at later developmental stages.

### Regulation of paralogous target genes by paralogous Cdx genes

The three Cdx paralogues were generated by the 2R WGD events at the origin of vertebrates. Similar duplicate genes dating to these genome duplications are called ‘2R-ohnologues’ or 2R-paralogues [40]. If sub-functionalization of genetic pathways after whole genome duplication took place, we would expect to see distinct regulatory interactions between sets of 2R paralogues at different levels within genetic pathways. We therefore examined the genes affected by Cdx MOs for cases where Cdx paralogues are regulating distinct paralogous target genes generated by the same genome duplication events.

We generated a candidate catalogue of 2R-paralogues using a reciprocal best-hits strategy between the amphioxus and *Xenopus* gene models, validated by phylogenetic reconstruction and examination of syntenic context. For example, we find that *Smad4.1* and *Smad4.2* are 2R paralogues with different responses to Cdx paralogues. Disruption of *Cdx2* function down-regulated *Smad4.2* expression, while disruption of *Cdx4* function up-regulated *Smad4.1* (Fig. 5g). Similar patterns were detected for several duplicated gene families encoding components of intracellular signalling pathways. Among these are the *phlpp1* and *phlpp2* proteins involved in dephosphorylation and inactivation of the Akt group of tyrosine kinases [41] and the *Presenilin* genes encoding core components of the gamma-secretase complex that cleaves and activates Notch, Erbb4, and other transmembrane proteins [42] (Fig. 5g). Among homeobox genes, three of the four *X. tropicalis Tlx* genes (*Tlx1*, *Tlx2*, *Tlx3*) were generated by the 2R genome duplications and are also differentially regulated by the duplicate Cdx genes (Fig. 5g). Some duplicated target genes involved in biochemical processes are also differentially regulated. The two paralogous selenophosphate synthetase genes, *Sephs1* and *Sephs2*, are differentially responsive to *Cdx4* and *Cdx1*, as are the two vertebrate-specific duplicates of phospholipase A2 group 12 *Pla2g12b* and *Pla2g12a*. Though it is difficult to quantitatively assess the extent of such ‘paralogous regulation’, the identification of these examples demonstrates that vertebrate genome duplications can generate distinct duplicated pathways of interacting genes.

## Discussion

### Redundancy versus complementation

The generation of three Cdx genes dates back over 500 million years, and in no vertebrate species analysed to date has any of these three genes been lost, with the exception of the teleost fish (which still have three Cdx genes, having two *Cdx1* but no *Cdx2* gene; [15]). One possible explanation for evolutionary retention of the three Cdx genes is that they have distinct roles, none of which can be dispensed with. Paradoxically, however, studies in several species have suggested rather similar roles for the three genes. Most noticeably, all are expressed in the posterior part of the embryo, including but not restricted to the posterior endoderm [22, 43, 44]. A similar Cdx expression pattern was also seen for the single, pre-duplication, cephalochordate Cdx gene [14], and most likely represents the ancestral condition inherited by vertebrates.

These observations do not rule out different roles, however, including activation or repression of the different developmental pathways needed for posterior body development. For instance, one important role for Cdx genes is activation of posterior Hox genes, ensuring correct patterning of the axial skeleton. Which Cdx genes are most important for these functions remains unclear, although it is notable that in mouse development *Cdx1* and *Cdx2* cooperate in Hox-mediated axial skeleton patterning with overlapping, though not fully redundant roles [18, 19].

In addition to roles in posterior and axial patterning, an alternative reason for retention of duplicate genes could be acquisition of new gene-specific roles. Cdx genes are essential for the proper development of gut epithelium, but again there is evidence in mice for active cooperation between *Cdx1* and *Cdx2* in this process [20]. In mammals, one of the Cdx genes, *Cdx2*, has been recruited for an additional role in specification of trophectoderm [45], but since this tissue is mammal-specific this does not help resolve why all three genes were retained through vertebrate evolution. Finally, Cdx genes are also involved in haematopoiesis but again there is evidence for functional overlap, with all three genes implicated in mice and at least two in zebrafish [17, 21, 27]. Therefore, most case studies point toward cooperation and overlapping roles of Cdx genes rather than acquisition of distinctly different roles during development.

### Quantitative and qualitative differences between Cdx genes

In this study, we attempted to tease apart to what extent Cdx genes have shared versus gene-specific roles in *Xenopus* development. A particularly striking finding is that disruption of *Cdx4* gene activity has a massively greater effect on downstream gene expression than caused by disruption of either *Cdx1* or *Cdx2*. Between 7 and 10 times more downstream genes are affected by interference with *Cdx4* gene function than by interfering with *Cdx1* or *Cdx2*. This effect is not attributable to efficiency of *Cdx4* disruption, since global comparison of transcriptome change (Fig. 2), clustering based on expression fold-change (Fig. 3), and pathway analyses (Fig. 4) each reveal qualitative differences between the three genes, generally with *Cdx4* as the outlier from *Cdx1* and *Cdx2*. Despite quantitative and qualitative differences, we also find examples of shared biological roles, including effects on Wnt signalling, genes involved in angiogenesis or digestive tract development, and a common role in modulating gene expression during the early phase of zygotic transcription after the MBT. Even in these examples, however, our data suggest that the *Cdx4* gene plays the dominant role amongst a set of overlapping paralogues in *X. tropicalis*. It is perhaps relevant that the *Cdx4* gene has been retained throughout vertebrate evolution, whereas *Cdx2* was lost in teleost fish perhaps replaced by the second *Cdx1* gene that arose in teleosts [15]. It is possible, therefore, that *Cdx4* diverged in function from *Cdx1* and *Cdx2* early in vertebrate evolution; this could be tested by comparing the effects of gene disruption on transcriptomes in zebrafish and *Xenopus*.

Response-based gene clustering and functional category enrichment also revealed groups of genes with distinct responsiveness to different Cdx genes. These include sets of genes that respond to *Cdx4* only, to *Cdx4* plus *Cdx1*, or to *Cdx1* plus *Cdx2*. Developmental pathways also show differences in responsiveness, including PDGF and heme biosynthesis pathways affected by *Cdx1* and *Cdx2* disruption, and *Cdx4* primarily regulating Hh, gonadotropin, and Slit/Robo axon guidance pathways. These include some previously unsuspected roles for Cdx genes. These differences suggest that *Cdx4* may have gene-specific roles within the posterior nerve cord, with both *Cdx1* and *Cdx2* functioning during haematopoiesis. In general, gene-specific roles could represent either ancestral roles that have been partitioned between paralogous genes after duplication (for example, by duplication-degeneration-complementation [5]) or they could be novel roles added to individual Cdx genes after duplication. These are fundamentally different ways in which gene regulatory networks could be rewired after WGD, as discussed later.

### Colinear effects on Hox genes by Cdx genes

The developmental role of Cdx genes in elongation of the body axis and positional specification is mediated principally through activation of posterior Hox genes [14, 19, 29]. This interaction was confirmed in the present study, where disruption of Cdx gene function (notably *Cdx4* and the Cdx triple MO) resulted in reduced expression of middle and posterior Hox genes (excluding paralogy groups 12 and 13 due to their later expression). An opposite effect is observed for anterior Hox genes, which are up-regulated by disruption of Cdx function (Fig. 5a–c). The relationship between physical position in a Hox cluster and effect of Cdx gene disruption could be a manifestation of colinearity, meaning that a biological property changes concomitantly with genomic position. The finding that Cdx genes affect Hox gene expression in an approximately colinear manner is novel, as is the finding that anterior and middle/posterior genes can be affected in opposite directions.

This leads us to propose Cdx-Hox gene interaction as a sliding scale, moving from mild Cdx repression of anterior Hox genes (Hox1 to Hox2) to strong Cdx activation of posterior Hox genes (Hox9 to Hox11). This effect could be mediated by a direct effect of Cdx proteins on remodelling chromatin state, or direct transcriptional control, although such an inference would need testing at the tissue or cellular level. Furthermore, any such mechanism would be different from the effect reported recently during cell differentiation [46], which involved only particular Hox genes. All four Hox gene clusters are similarly affected, implying that the establishment of putative colinear regulation of Hox genes by Cdx genes predates the 2R genome duplications and the origin of vertebrates.

### Rewiring gene regulatory networks after WGD

WGD is expected to be a powerful evolutionary force, as simultaneous duplications of many different genes provides a great opportunity to reshuffle the interactions within gene regulatory networks [13]. Multiple regulatory schemes could arise after duplication, namely one gene regulating two duplicated targets, two duplicate genes regulating one target, or two duplicate genes each regulating different duplicated targets. We detected several such examples of elaborated post-duplication regulatory reshuffling in our dataset. The six clearest examples are the co-factor Smad genes, the Phlpp genes, the Presenilin genes, the Tlx genes, the selenophosphate synthetase genes, and the phospholipase A2 group 12 genes (Fig. 5f). Most of these examples comprise genes critically involved in developmental processes, such as intercellular communication, intracellular signalling, or activation of cell-type specific gene expression, which is reminiscent of previous results stressing the over-representation of such functional categories in post-duplication retained genes [10].

Each of these cases is likely to have evolved from an ancestral regulatory role of the Cdx gene, before genome duplication, with the paralogous Cdx and target genes elaborating on the ancestral interaction. However, reconstructing the full ancestral (pre-duplication) target set from our dataset is not possible. Some ancestral targets are likely to be present in the core set of 177 genes co-regulated by two or more vertebrate Cdx genes, but there may be other ancestral targets now regulated by only a single Cdx gene. A powerful way to distinguish pre-duplication and post-duplication targets would be by comparison to Cdx target genes in non-vertebrates; however, for this, functional interference techniques will be needed in systems such as annelids, echinoderms, and cephalochordates [47]. It would also be informative to examine empirically-determined genetic or protein-protein interaction networks [48] for 2R paralogues for traces of gene network evolution following genome evolution.

Two alternative models could explain the maintenance of paralogues after WGD: the first one proposes that paralogues largely share overlapping functions, which renders their presence necessary for downstream function through either dosage or through developmental buffering; the alternative model postulates that sub-functionalization and functional divergence redistribute the role of each paralogue, which makes their roles non-overlapping, but similarly essential. Our present dataset provides evidence that both these mechanisms are at play, but rejects that either of them could accurately predict the rewiring of genetic pathways after WGD.

## Conclusions

Herein, we have interfered with the function of all members of a developmentally important homeobox gene family and, for the first time, analysed detailed responses at the transcriptomic level. Our results show that duplicated Cdx genes share some common sets of target genes and downstream biological pathways, but there are also significant differences between the three genes. In development of *X. tropicalis*, each Cdx gene is responsible for orchestrating a unique transcriptome profile during development. These differences are manifest both quantitatively and qualitatively, with the *Cdx4* gene playing a quantitatively larger role than *Cdx1* and *Cdx2* in this species. These results suggest that the extent of functional redundancy between duplicated developmental regulators in vertebrates could have been overestimated, with shared roles and expression domains masking important functional differences manifest at the transcriptional level. We suggest that inherent robustness of vertebrate developmental and morphogenetic processes, with multiple pathways converging to generate complex phenotypes, can mask distinctive molecular phenotypes associated with different WGD paralogues. More generally, these data reveal that after WGD in the early evolution of vertebrates, paralogous genes encoding key transcriptional regulators maintained some shared roles yet also diverged functionally in evolution to orchestrate developmental complexity and robustness.

## Methods

### Morpholino injection

*X. tropicalis* eggs were obtained and fertilised as previously described in Faas L and Isaacs HV [23]. Staging followed Nieuwkoop PD and Faber J [49]. MOs to *Cdx1*, *Cdx2*, and *Cdx4* are described in Faas L and Isaacs HV [23] as ‘set-1’ and were designed by Amaya E. These MOs target the 5’UTR region of each mRNA and/or translation start site and block translation; specificity and effectiveness of translation inhibition has been verified previously by Western blotting, comparison of action to mismatch MOs, and rescue using an Xcad-VP16 construct [23]. For each MO experiment, 20 ng MO per embryo were injected at the 1- or 2-cell stage, in a total volume of 10 nL divided between each cell. When MOs targeted to all three Cdx genes were co-injected (‘triple’ or CdxA treatment), 6.67 ng of each MO were mixed. Two controls were also prepared: ‘Mock’ being injection of 20 ng of a standard control MO shown to have little discernible morphological effect [23] and ‘Back’ (background) comprising uninjected embryos. Both were required since gene expression changes can result from injection trauma or exposure to foreign molecules. Three replicate experiments were performed, at separate times, and using eggs and sperm from different parents; in each case the six conditions (Cdx1 MO, Cdx2 MO, Cdx4 MO, Triple MOs, Mock MO, Back) were performed on batches of sibling embryos; 10 to 20 embryos were harvested at early neurula stage 14 and total RNA prepared by the methods of Branney et al. [50].

### Transcriptome characterisation

mRNAseq libraries were prepared for each RNA sample using the TruSeq RNA kit (Illumina) at the Oxford Genomics Centre in the Wellcome Trust Centre for Human Genetics, University of Oxford. This procedure involves isolation of polyadenylated transcripts, chemical fragmentation, randomly primed reverse-transcription, and adapter ligation. Six libraries from a biological replicate were pooled on the same HiSeq2000 lane. Sequencing was performed for 100 cycles in paired-end mode, resulting in approximately 32 million paired-end reads for each sample (ranging between 25.2 M and 43.6 M reads per sample). Reads from each library were mapped to the JGI 4.2 version of the *X. tropicalis* genome (downloaded from [51]) using the splice-aware aligner TopHat (v2.0.4) with Ensembl gene models as guides for alignment [52]. The average mapping rate across libraries was 86 %, the value for each library ranging between 60 % and 92 %. We obtained read counts from Ensembl gene models using HTSeq library with an average 58.5 % of mapped reads within annotated exons [53]. In total, 18,336 of the 19,884 Ensembl gene models were covered by our transcriptome data in at least one sample. RNA-seq raw data and read counts have been deposited in the NCBI Gene Expression Omnibus (www.ncbi.nlm.nih.gov/geo) under accession GSE71006.

### Differential expression

Differential expression analyses were conducted using DESeq2 [54]. The biological variation between replicates is relatively strong, possibly due to genetic differences between batches of embryos, subtle staging differences, or physiological effects (Additional file 7: Figure S1). To compensate for such effects, we accounted explicitly for the paired nature of replicates using a multifactor design [54]. The model estimates the replicate effect associated with the paired nature of the samples, and improves detection of the treatment effect. Morpholino-injected samples were compared against the ‘Mock’ condition. Subsequently, genes showing significant differential expression in the ‘Mock’ against ‘background’ were excluded as potentially affected by injection (Additional file 1: Table S1). To support diagnostics in differential expression, FPKM values (fragment per kilobase and per million reads) were calculated for the Ensembl gene models using Cuffnorm in the Cufflinks package [55]. Several criteria were used to delineate a confident set of differentially expressed genes in each condition: (1) an adjusted *P* value (Benjamini-Hochberg correction) lower than 0.05 for the condition of interest, (2) unaffected in the ‘Mock’ against ‘background’ comparison (adjusted *P* >0.05), (3) an expression level greater than 2 FPKMs, and (4) a fold-change between reference and condition greater than 1.41 (log_2_(Fold-change) >0.5). Additional file 1: Table S1 gives the numbers of differentially expressed genes retained after filtering with each criterion. Additional file 8: Table S4 gives the differential expression statistics for all genes significant in at least one condition.

### Functional annotation

Gene ontology terms and Panther pathway annotation was extracted from Panther 9.0 (downloaded from [56]). Gene Set Enrichment Analysis was performed in R using the Piano 1.4 package [57]. Enrichment was scored assuming either non-directionality (disregarding direction of fold-change) or directionality (up- and down-regulated genes cancel out) and the best scoring method was retained for each functional category.

Clustering was employed to detect groups of genes with similar expression profiles across conditions. We selected the 2,683 genes showing significant differential expression in at least one condition and applied clustering on their fold-change values using Euclidean distances and Ward’s agglomeration method. We distinguished 12 clusters (k) that best recapitulate the diversity of expression profiles across conditions.

To obtain temporal expression profiles for differentially expressed genes, we deployed stage-specific RNAseq data from Tan et al. [25] (accession: GSE37452). These read data were extracted, mapped to the *X. tropicalis* genome, and read counts for Ensembl gene models recovered as described above. For each gene, expression values were normalized relative to their time of maximum expression. We then performed clustering according to temporal expression profiles using just the 2,683 genes that show differential expression in at least one condition. The frequencies of the seven clusters found to best recapitulate the expression profiles were examined among differentially expressed genes in all conditions.

### Ohnologue analysis

We first identified candidate sets of *X. tropicalis* genes putatively originating through the 2R vertebrate genome duplications by performing reciprocal blast between Ensembl *Xenopus* gene models and *Branchiostoma floridae* predicted proteins. We found 6,198 *Xenopus* genes satisfying an orthology relationship of 2, 3, or 4 to 1 with 1,255 amphioxus genes. These putative paralogy groups include 496 *Xenopus* genes differentially expressed in at least one morpholino condition. We retained the 32 cases in which at least two genes are differentially expressed in two distinct conditions. These candidates were further filtered by examining chromosomal position in human and *X. tropicalis* genomes for evidence of location in known 2R paralogy regions, and through phylogenetic analysis to date gene duplication events.

## Additional files

Additional file 1: Table S1. Number of differentially expressed genes after sequential cut-offs. (DOCX 47 kb) Additional file 2: Figure S2. Relationship between adjusted *P* values and expression fold-change in differential expression (‘volcano’ plot). (PDF 5370 kb) Additional file 3: Table S2. GO BP and Panther term enrichment in differentially expressed gene sets exclusive to the specific conditions (*Cdx1*, *Cdx2*, *Cdx4*, and Triple). Remaining genes regulated by two or more Cdx genes are grouped as ‘2ormore’. (XLSX 12 kb) Additional file 4: Table S3. GO BP and Panther term enrichment in clusters (corresponding to that of Fig. 3). (XLSX 14 kb) Additional file 5: Figure S3. Effect of morpholino oligonucleotides (MO) injection for each Cdx paralogue on Hox gene expression in *X. tropicalis*. Significant effects on Hox gene expression are indicated by a plain border, whereas non-significant effects are marked with a dotted border. The top right graph shows fold-change in Hox gene expression caused by triple MO injection plotted against paralogy group assignment; each data point represents one Hox gene. All genes included. Colours denote anterior (red), middle (green), and posterior (blue) paralogy group assignments, assigning group 3 to anterior. The bottom right graph shows expression fold-change compared between grouped sets of anterior, middle, and posterior genes (all four clusters, all Hox genes). (PDF 386 kb) Additional file 6: Figure S4. Expression fold-change of selected target genes belonging to Wnt and FGF gene families. The (*) indicates genes significant in differential expression analysis. (PDF 383 kb) Additional file 7: Figure S1. Heatmap representation of log-transformed normalised read counts in replicates of the experimental conditions for the 500 most expressed genes. As discussed, replicate 1 is divergent from the others. (PDF 350 kb) Additional file 8: Table S4. List of genes with differential expression triggered by Cdx MOs as determined by DESeq2. (XLSX 578 kb)

## Abbreviations

2R: Two rounds
FGF: Fibroblast growth factor
MBT: Mid-blastula transition
MO: Morpholino oligonucleotide
WGD: Whole genome duplication
