# Supplementary material for: Cdx ParaHox genes acquired distinct developmental roles after gene duplication in vertebrate evolution
Source: BMC Biol. 2015 Aug 1;13:56. doi: 10.1186/s12915-015-0165-x (PMC4522105; doi:10.1186/s12915-015-0165-x)
Supplement: Additional file 1: Table S1. — Number of differentially expressed genes after sequential cut-offs. (DOCX 47 kb) [file 12915_2015_165_MOESM1_ESM.docx]

**Supplementary Table S1.** Number of differentially expressed genes after sequential cut-offs

|  | **Cdx1** | **Cdx2** | **Cdx4** | **Triple** | **Back-Mock** |
| --- | --- | --- | --- | --- | --- |
| p_MO_ <0.05 | 602 | 461 | 3471 | 1919 | 158 |
| p_MO_ <0.05 & FPKM>2 | 580 | 447 | 3083 | 1763 | 153 |
| p_MO_ <0.05 & FPKM>2 & p_BM_>0.05 | 513 | 361 | 2988 | 1682 | - |
| p_MO_ <0.05 & FPKM>2 &  p_BM_>0.05 & log_2_FC>0.5 | 304 | 213 | 2018 | 1132 | - |

p_MO_ is the p-value of the test for condition effect, and p_BM_ is the p-value for ‘background’ vs. ‘Mock’ test corresponding to genes likely induced by MO injection.
