# Supplementary figures and images for: Cdx ParaHox genes acquired distinct developmental roles after gene duplication in vertebrate evolution
Source: BMC Biol. 2015 Aug 1;13:56. doi: 10.1186/s12915-015-0165-x (PMC4522105; doi:10.1186/s12915-015-0165-x)

**Cdx1**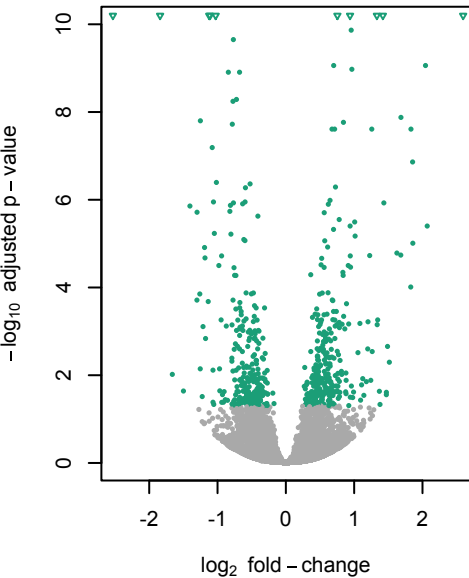**Cdx2**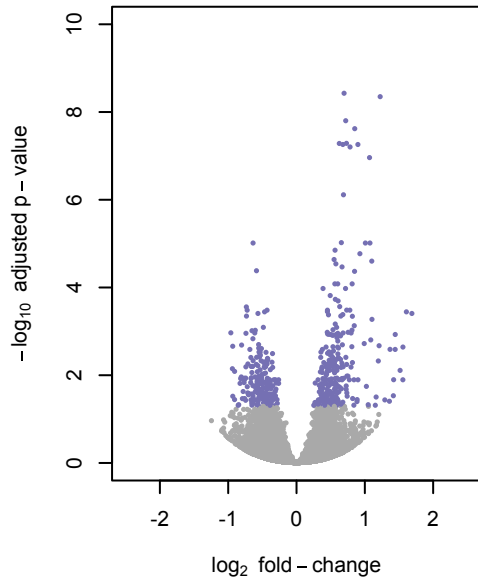**Cdx4**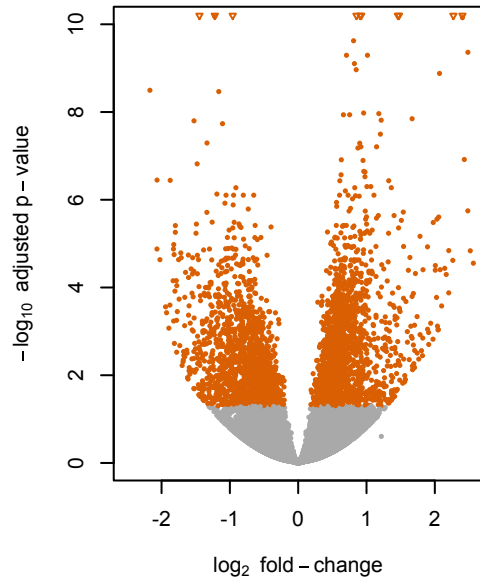

Supplement: Additional file 2: Figure S2. — Relationship between adjusted P values and expression fold-change in differential expression (‘volcano’ plot). (PDF 5370 kb) [file 12915_2015_165_MOESM2_ESM.pdf]

Paralogy groups (PG)

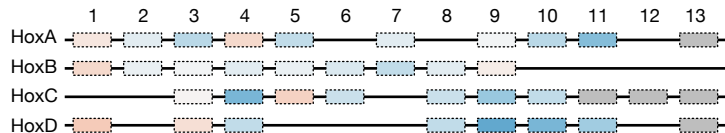

**Cdx1**

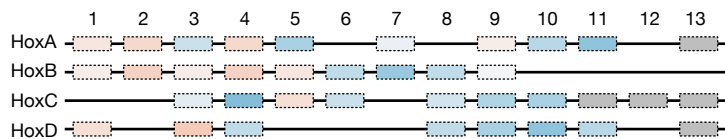

**Cdx2**

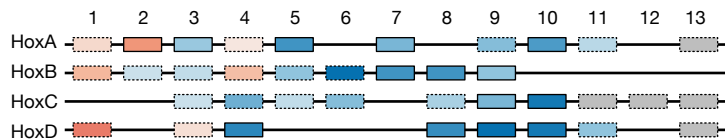

**Cdx4**

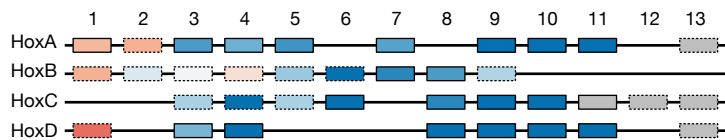

**Triple**

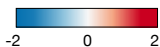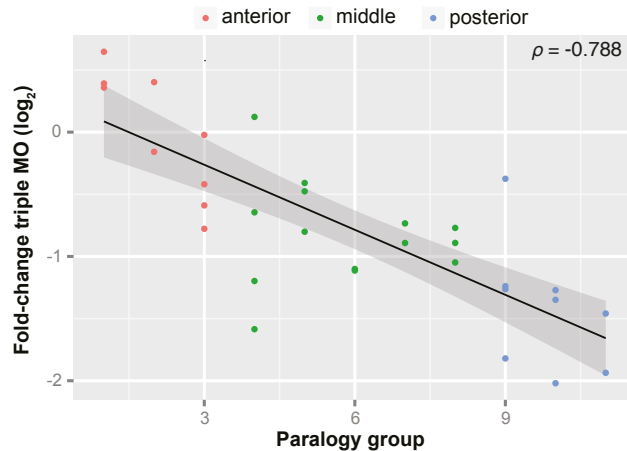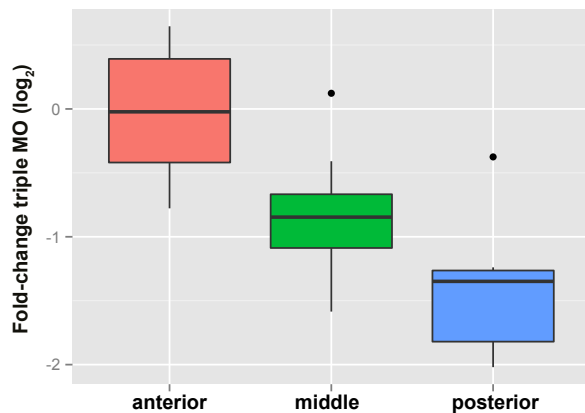

Supplement: Additional file 5: Figure S3. — Effect of morpholino oligonucleotides (MO) injection for each Cdx paralogue on Hox gene expression in X. tropicalis. Significant effects on Hox gene expression are indicated by a plain border, whereas non-significant effects are marked with a dotted border. The top right graph shows fold-change in Hox gene expression caused by triple MO injection plotted against paralogy group assignment; each data point represents one Hox gene. All genes included. Colours denote anterior (red), middle (green), and posterior (blue) paralogy group assignments, assigning group 3 to anterior. The bottom right graph shows expression fold-change compared between grouped sets of anterior, middle, and posterior genes (all four clusters, all Hox genes). (PDF 386 kb) [file 12915_2015_165_MOESM5_ESM.pdf]

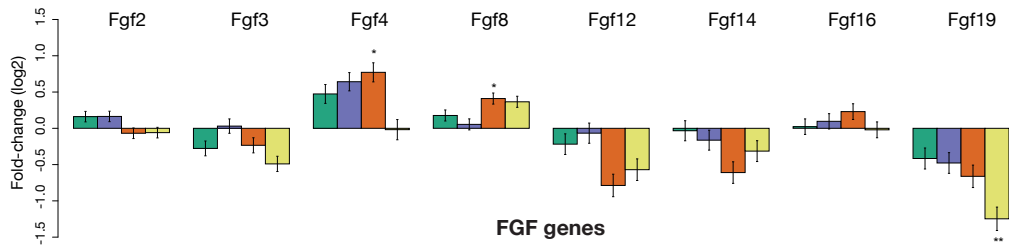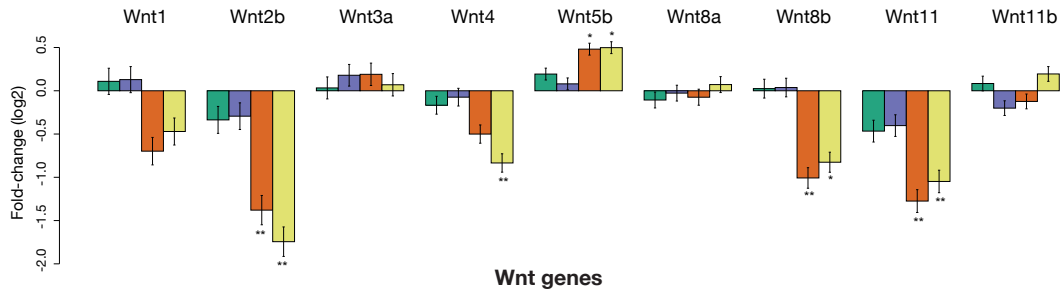

Supplement: Additional file 6: Figure S4. — Expression fold-change of selected target genes belonging to Wnt and FGF gene families. The (*) indicates genes significant in differential expression analysis. (PDF 383 kb) [file 12915_2015_165_MOESM6_ESM.pdf]

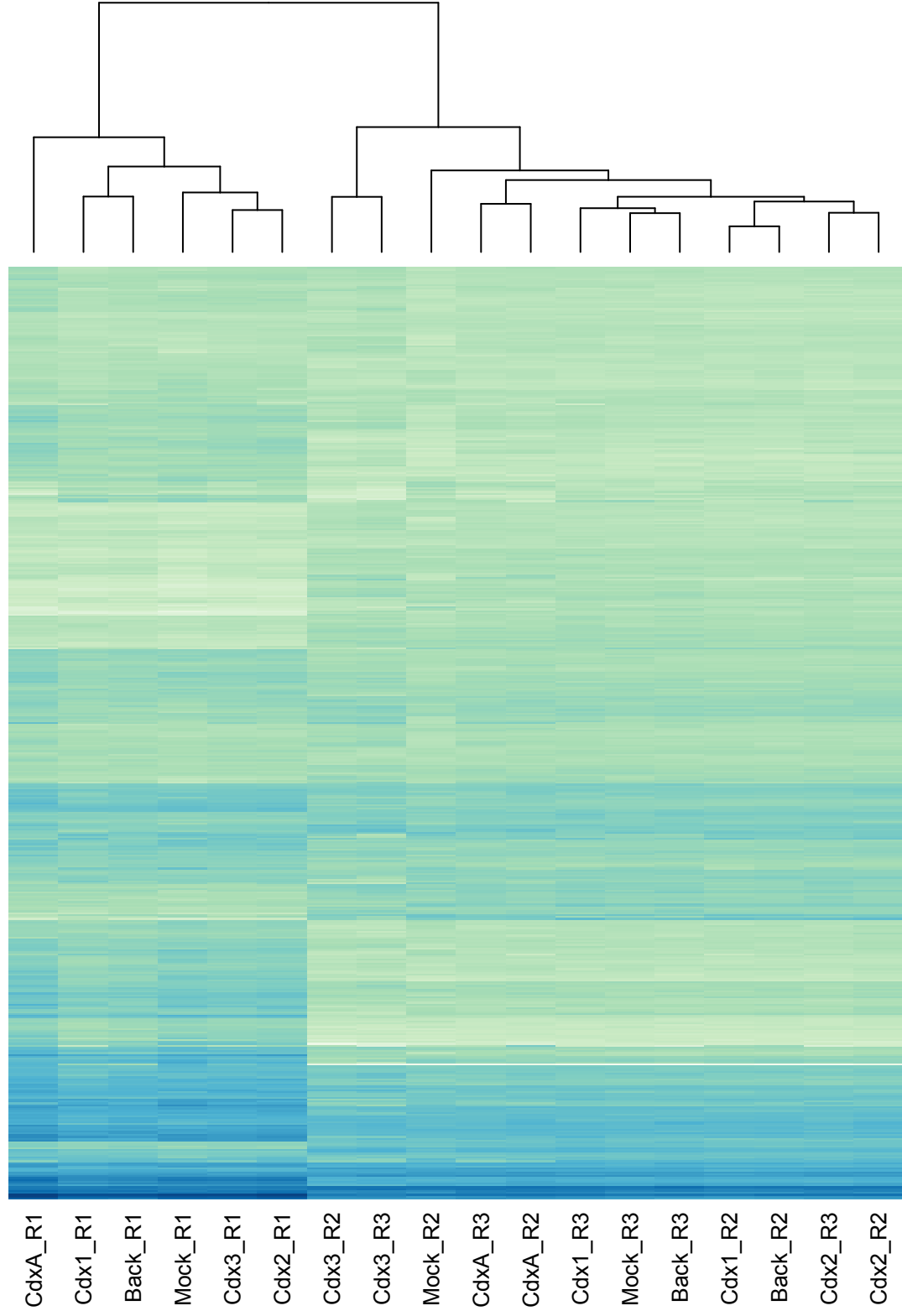

Supplement: Additional file 7: Figure S1. — Heatmap representation of log-transformed normalised read counts in replicates of the experimental conditions for the 500 most expressed genes. As discussed, replicate 1 is divergent from the others. (PDF 350 kb) [file 12915_2015_165_MOESM7_ESM.pdf]
